# Disparities in access to hematopoietic cell transplant persist at a transplant center

**DOI:** 10.1038/s41409-024-02327-x

**Published:** 2024-06-13

**Authors:** Jamie Shoag, Seth J. Rotz, Rabi Hanna, Ilia Buhtoiarov, Elizabeth N. Dewey, David Bruckman, Betty K. Hamilton

**Affiliations:** 1 Division of Pediatric Hematology, Oncology, and Blood & Marrow Transplantation, Pediatric Institute, Cleveland Clinic, Cleveland, OH USA; 2 Center for Populations Health Research, Department of Quantitative Health Sciences, Cleveland Clinic, Cleveland, OH USA; 3 Department of Hematology and Medical Oncology, Blood and Marrow Transplantation, Cleveland Clinic, Taussig Cancer Institute, Cleveland, OH USA

**Keywords:** Health services, Public health

## Abstract

Disparities in access to hematopoietic cell transplant (HCT) are well established. Prior studies have identified barriers, such as referral and travel to an HCT center, that occur before consultation. Whether differences in access persist after evaluation at an HCT center remains unknown. The psychosocial assessment for transplant eligibility may impede access to transplant after evaluation. We performed a single-center retrospective review of 1102 patients who underwent HCT consultation. We examined the association between race/ethnicity (defined as Hispanic, non-Hispanic Black, non-Hispanic White, and Other) and socioeconomic status (defined by zip code median household income quartiles and insurance type) with receipt of HCT and Psychosocial Assessment of Candidates for Transplantation (PACT) scores. Race/ethnicity was associated with receipt of HCT (*p* = 0.02) with non-Hispanic Whites comprising a higher percentage of HCT recipients than non-recipients. Those living in higher income quartiles and non-publicly insured were more likely to receive HCT (*p* = 0.02 and *p* < 0.001, respectively). PACT scores were strongly associated with income quartiles (*p* < 0.001) but not race/ethnicity or insurance type. Race/ethnicity and socioeconomic status impact receipt of HCT among patients evaluated at an HCT center. Further investigation as to whether the psychosocial eligibility evaluation limits access to HCT in vulnerable populations is warranted.

## Background

Hematopoietic cell transplant (HCT) is a curative therapy for many malignant and non-malignant diseases [1]. With advancements in conditioning regimens and donor sources, the number of patients eligible to receive HCT in the United States is increasing [2]. However, racial, ethnic, and socioeconomic disparities continue to limit access to patients who would otherwise benefit [1–4].

**Fig. 1:**
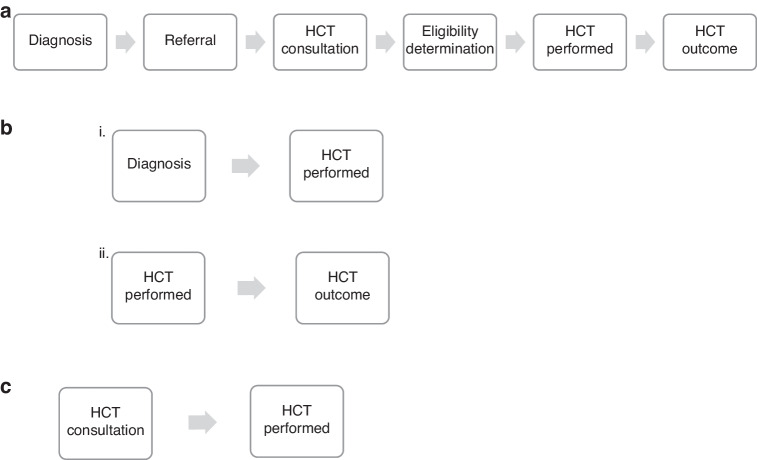
Progression map to hematopoietic cell transplant (HCT). **a** Patient journey through HCT. **b** Focus of prior research studies. **c** Focus of this study.

Prior studies have established marked disparities in access to HCT [5–8]. However, there is limited knowledge as to where in the journey from diagnosis to transplant inequities occur (Fig. 1). Thus far, identified barriers, such as referral [9] and travel [10] to a bone marrow transplant center, occur prior to HCT evaluation [2]. Nevertheless, even amongst those referred for consultation, bias may interfere with the receipt of a transplant via the eligibility determination.

The HCT eligibility determination is a multi-disciplinary decision that weighs the risk of morbidity and mortality associated with HCT compared with those of alternative treatment options [11]. Eligibility assessments consider several disease-, patient-, and donor-related factors that influence the anticipated success of transplant [12]. The National Marrow Donor Program also recommends a comprehensive psychosocial assessment that considers any issues that would adversely influence transplant outcomes [12]. The goal of the psychosocial assessment is to ensure patients have the necessary support to succeed both during and after the HCT process [13]. Due to a lack of prospective data or comprehensive consensus guidelines, psychosocial eligibility assessments vary across transplant centers and determination is made on a case-by-case basis [11, 12, 14].

The Psychosocial Assessment of Candidates for Transplantation (PACT) score is validated and routinely used psychosocial evaluation in solid organ transplantations (SOT) given a limited supply of organs [15]. PACT is an 8-item rating scale addressing social support, psychological health, lifestyle factors, understanding of the transplant process, and support [16]. The final score (ranging from 0 to 4) also includes the assessor’s impression of patient compliance, substance abuse, and coping strategies [16]. Higher PACT scores (lower-risk patients) are associated with improved survival in SOT recipients [17]. However, data on the influence of PACT scores on HCT outcomes are conflicting. Some studies report an association between specific psychosocial factors with overall survival or secondary medical outcomes [18–21]. However, others found no association when controlling for transplant-related factors [18–22].

Here, we investigated the association of race, ethnicity, and socioeconomics with the likelihood of proceeding to HCT among candidates evaluated for transplant and differences in PACT scores among transplant recipients. This study was performed using the Cleveland Clinic Cancer Center Unified Transplant Database (UTD). The UTD is an institutional clinical research database of all patients evaluated for HCT, including those who do not proceed to transplant. Large national registries typically do not include detailed disease or patient-related information necessary to determine whether HCT consultation was indicated, while HCT registries collect comprehensive information on patients who receive HCT but do not have information on patients who do not [2]. Therefore, the UTD provides a unique opportunity to address a critical knowledge gap of potential disparities in receipt of HCT after consultation. Our hypothesis is that disparities in access to transplants exist even after HCT evaluation and that PACT scores differ among those transplanted.

## Methods

### Data source and cohort

A retrospective review of UTD records was performed under the guidance of the Cleveland Clinic’s Institutional Review Board. Informed consent for the collection of patient data was obtained in accordance with the Declaration of Helsinki. Pediatric and adult subjects who underwent consultation for HCT between January 1, 2015, and December 31, 2018, were included. Some patients had multiple consultations. For these subjects, a single record was selected consisting of the consultation date closest to the transplant date. For patients with multiple consultations who did not proceed to transplant, the most recent consultation record was selected.

Characteristics collected included age at consultation, sex (male or female), race, ethnicity, insurance status, median income quartile by zip code at the time of assessment, and vital status. Among patients who received HCT, PACT score, HCT-CI (hematopoietic cell transplant comorbidity index) score, Karnofsky performance score, and diagnosis were collected. All scores were assigned prior to transplant during the eligibility determination.

### Definitions

Race and ethnicity were self-reported by patients and obtained from registration data in the electronic medical record. Race and ethnicity data were used to create a 4-level “race/ethnicity” variable which included Hispanic (of any race), Non-Hispanic Black, Non-Hispanic White, and Other. This was done to allow explicit analysis of racial and ethnic identity as social constructs that serve as proxies for individual and collective disparities due to structural racism [23, 24]. Small sample sizes precluded modeling of other individual racial categories.

Socioeconomic status (SES) was proxied by insurance status and median income by zip code. Insurance status was dichotomized into public (including Medicare and Medicaid) and private/other insurance. Median income by zip code was obtained from the 2018 Census Table (5-year estimates from the American Community Survey) and linked to the patient zip codes. Postal codes from Canada and other areas were excluded. Median incomes were separated into quartile ranges found in the 2018 National Inpatient Survey data element definitions from the Healthcare Cost and Utilization Project [25]. Quartiles 1–4 reflect the poorest to wealthiest populations, respectively. Median income per zip code was chosen as the best measure since addresses were not available for geocoding and determination of the Area Deprivation Index [26].

PACT scores were analyzed ordinally from 0 through 4 as described above. HCT-CI scores were dichotomized into <3 (low/intermediate risk for non-relapse mortality) and ≥3 (high risk for non-relapse mortality). Karnofsky scores were dichotomized as ≤80 or >80. Common diagnoses were grouped into 4 categories including leukemia; myeloma/amyloidosis; myelodysplastic syndrome (MDS)/ myeloproliferative neoplasm (MPN)/ myelofibrosis; and lymphoma.

### Statistical analysis

Data was reported as frequencies and percentages. Central measures were presented as means ± standard errors or 95% confidence limits or as medians with 25th and 75th percentiles. For comparisons of means, medians, and categorical tests of association, we applied the Satterthwaite t-test or analysis of variance (ANOVA), Wilcoxon Rank Sum or Kruskal–Wallis test, Pearson’s or Fisher’s Exact chi-square test as appropriate. PACT values were compared across two-level factors using the Cochran–Mantel–Haenzel (CMH) test or the nonzero correlation test (NCT) with 1 degree of freedom. The CMH test for trend was applied to test an ordinal trend across PACT scores across other categorical factors; the CMH nonzero correlation test (NCT) requires both variables to be ordinal. Significance was set at *p* < 0.05 unless noted when adjusted for multiple comparisons.

## Results

### Cohort demographics

We identified a total of 1102 unique records of patients who underwent HCT consultation. The median age at consultation was 60.6 years (interquartile range [IQR] 50.5, 67.0 years). The cohort was 40.9% female. By race/ethnicity, the cohort was 2.3% Hispanic, 8.9% non-Hispanic Black, 85.5% non-Hispanic White, and 3.3% Other. Most patients (63.2%) had private/other insurance. By quartiles of median income for patients’ zip code, 22.1% were from quartile (Q) 1 ($1–45,999), 31.3% from Q2 ($46,000–58,999), 31.8% from Q3 ($59,000–78,999), and 14.9% from Q4 ($79,000+). At the time of data collection, 43.2% were deceased.

### Receipt of HCT

Table 1 shows the distribution of patients by receipt of HCT. Over half (59.5%) of patients had a transplant in the study period. Race/ethnicity was associated with receipt of transplant (*p* = 0.02) such that non-Hispanic White patients comprised a higher percentage of transplant recipients (87%) than non-recipients (83.3%). Neighborhood household income was associated with receipt of HCT (*p* = 0.02). Patients in the lowest income quartile accounted for 26.3% of those who did not receive HCT but only 19.2% of those receiving HCT. In contrast, patients in the highest income quartile accounted for 13% of those who did not receive HCT but 16.2% of those who received HCT. Insurance type was also associated with receipt of HCT (*p* < 0.001). Patients who were publicly insured accounted for 47.2% of those not transplanted but only 30.4% of those who were transplanted. Age was also associated with receipt of HCT (*p* < 0.001) with a higher median age among those who did not receive HCT (62.7, IQR 53.1–68.8) compared to those who received HCT (59.3, IQR 49.4–66.1). Notably, age and insurance were not observed to be confounded (interaction term *p* = 0.17) when an age and insurance type interaction term was included in a model testing receipt of HCT. More than half (52.6%) of patients who were assessed but did not receive HCT had died by the time data was collected. More than one-third (36.8%) of those assessed and receiving an initial transplant died between receipt of the transplant and data collection.

**Table 1:** Cohort demographics by receipt of HCT.

Factor	Total (*N* = 1102)	Did not receive HCT (*N* = 446)	Received HCT (*N* = 656)	*p*-value
**Age at most recent assessment**	60.6 [50.5, 67.0]	62.7 [53.1, 68.8]	59.3 [49.4, 66.1]	***<0.001***^a^
**Sex**				0.26^b^
Female	449 (40.9)	172 (38.8)	277 (42.2)	
Male	650 (59.1)	271 (61.2)	379 (57.8)	
Race/Ethnicity*				***0.02***^b^
Hispanic, any race	25 (2.3)	7 (1.6)	18 (2.7)	
Non-Hispanic Black	97 (8.9)	44 (10.1)	53 (8.1)	
Non-Hispanic White	934 (85.5)	363 (83.3)	571 (87.0)	
Other	36 (3.3)	22 (5.0)	14 (2.1)	
**Insurance Type**				***<0.001***^b^
Public (Medicare/caid)	364 (36.8)	177 (47.2)	187 (30.4)	
Private/Other	625 (63.2)	198 (52.8)	427 (69.5)	
**Zip code median income quartile (Q)**				***0.02***^a^
Q1 ($1–45,999)	233 (22.1)	113 (26.3)	120 (19.2)	
Q2 ($46,000–58,999)	330 (31.3)	128 (29.8)	202 (32.3)	
Q3 ($59,000–78,999)	335 (31.8)	133 (30.9)	202 (32.3)	
Q4 ($79,000+)	157 (14.9)	56 (13.0)	101 (16.2)	
**Patient Deceased**				***<0.001***^b^
No	623 (56.8)	209 (47.4)	414 (63.2)	
Yes	473 (43.2)	232 (52.6)	241 (36.8)	

Statistics presented as Median [P25, P75], *N* (column %).

Statistically significant *p*-values are in bold.

*Data not available for all subjects. Missing values: Race/Ethnicity = 10.

*p*-values:

^a^Wilcoxon Rank Sum test.

^b^Pearson’s chi-square test.

### PACT scores

Ordinal PACT scores by patient demographics are shown in Table 2. Higher PACT scores were associated with older median age at assessment (*p* = 0.09). PACT scores did not differ in distribution by race/ethnicity (CMH test for trend *p* = 0.45) or insurance type (CMH test for trend *p* = 0.10). However, a strong bias was observed between median income quartiles and ordinal PACT scores (NCT for trend *p* < 0.001, *p* = 0.009 overall association). Higher PACT scores were strongly associated with higher median income for the patient zip code and lower PACT scores were strongly associated with lower median income for the patient zip code.

**Table 2:** Ordinal trends of PACT scores among HCT recipients by demographics.

Factor	PACT score				*p*-value
	1	2	3	4	
**Age at most recent assessment**	49.7 [36.8, 50.1]	57.2 [48.4, 64.0]	59.9 [49.9, 65.9]	60.3 [49.9, 67.1]	0.09^d^
**Sex**					0.45^b^
Female	0 (0)	24 (9.9)	149 (61.6)	69 (28.5)	
Male	1 (0.3)	37 (11.5)	192 (59.6)	92 (28.6)	
**Race/Ethnicity**					0.45^b^
Hispanic, Any Race	0 (0)	0 (0)	8 (66.7)	4 (33.3)	
Non-Hispanic Black	0 (0)	8 (18.6)	27 (62.8)	8 (18.6)	
Non-Hispanic White	1 (0.2)	52 (10.4)	302 (60.4)	145 (29.0)	
Other	0 (0)	1 (11.1)	4 (44.4)	4 (44.4)	
**Insurance type**					
Public (Medicare/caid)	0 (0)	26 (14.9)	103 (59.2)	45 (25.9)	0.10^b^
Private/Other	1 (0.2)	36 (9.1)	242 (61.0)	118 (29.7)	
**Zip code median income quartile (Q)**					
Q1 ($1–45,999)	0 (0)	26 (23.1)	62 (54.9)	25 (22.1)	***<0.01***^a^ < ***0.001***^c^
Q2 ($46,000–58,999)	2 (1.1)	25 (13.3)	111 (59.0)	50 (26.6)	
Q3 ($59,000–78,999)	1 (0.5)	16 (8.2)	122 (62.6)	56 (28.7)	
Q4 ($79,000+)	0 (0)	8 (8.3)	54 (56.2)	34 (35.4)	
**Patient deceased**					
No	2 (0.5)	48 (12.4)	223 (57.8)	113 (29.3)	0.59^b^
Yes	1 (0.4)	23 (10.3)	146 (65.2)	54 (24.1)	

Data presented on HCT recipients. PACT data not available for all subjects. Statistics presented as median [interquartile range, IQR] or N (row %).

Statistically significant *p*-values are in bold.

Categorical tests rechecked for significance excluding PACT score = 1.

*p*-values:

^a^Pearson’s chi-square test.

^b^Cochran–Mantel–Haenszel chi-square ordinal test for trend.

^c^CMH nonzero correlation test (NCT) for ordinal trend.

^d^Kruskal–Wallis test.

### HCT-CI scores, Karnofsky performance sores, and diagnoses

To assess whether physical health differed by demographics, we investigated differences in baseline HCT-CI and Karnofsky performance scores (Tables 3 and 4, respectively). There was no difference in HCT-CI score ≥3 by age (*p* = 0.28), sex (*p* = 0.54), race/ethnicity (*p* = 0.28), insurance type (*p* = 0.07), or median income quartile (*p* = 0.24). There was no difference in Karnofsky performance score >80 by sex (*p* = 0.28), race/ethnicity (*p* = 0.29), or median income quartile (*p* = 0.23). Karnosky score differed by age (*p* = 0.09) and insurance status (p < 0.001) such that a younger median age at assessment and private/other insurance was associated with Karnofsky performance score >80.

**Table 3:** HCT-CI scores among HCT recipients by demographics.

Factor	Total (*N* = 636)	HCT-CI < 3 (*N* = 307)	HCT-CI ≥ 3 (*N* = 329)	*p*-value
Age at most recent assessment*	59.5 [49.7, 66.3]	59.2 [49.0, 65.8]	59.9 [49.9, 66.4]	0.28^a^
Sex				0.54^b^
Female	268 (42.1)	133 (43.3)	135 (40.9)	
Male	369 (57.9)	174 (56.7)	195 (59.1)	
Race/Ethnicity*				0.28^b^
Hispanic, any race	17 (2.7)	6 (2.0)	11 (3.3)	
Non-Hispanic Black	50 (7.9)	20 (6.5)	30 (9.1)	
Non-Hispanic White	555 (87.3)	276 (89.9)	279 (84.8)	
Other	14 (2.2)	5 (1.6)	9 (2.7)	
Insurance Type*				0.07^b^
Public (Medicare/caid)	183 (30.7)	80 (27.2)	103 (34.1)	
Private/Other	413 (69.3)	214 (72.8)	199 (65.9)	
Zip code median income quartile (Q)				0.24^b^
Q1 ($1–45,999)	114 (18.8)	47 (16.2)	67 (21.3)	
Q2 ($46,000–58,999)	194 (32.0)	90 (30.9)	104 (33.0)	
Q3 ($59,000–78,999)	199 (32.8)	105 (36.1)	94 (29.8)	
Q4 ($79,000+)	99 (16.3)	49 (16.8)	50 (15.9)	

Data presented on HCT recipients. HCT-CI score data not available for all subjects.

*Data not available for all subjects. Missing values: Race/Ethnicity = 10; Insurance Type = 40; PACT score = 32.

Statistics presented as *N* (column %).

*p*-values:

^a^Wilcoxon Rank Sum test.

^b^Pearson’s chi-square test.

**Table 4:** Karnofsky performance scores among HCT recipients by demographics.

Factor, *n* (%)	Total (*N* = 648)	Karnofsky > 80 (*N* = 476)	Karnofsky ≤ 80 (*N* = 172)	*p*-value
**Age at most recent assessment**	59.4 [49.4, 66.1]	58. [48.7, 66.1]	60.6 [53.3, 66.0]	0.09^a^
**Sex**				0.28^b^
Female	275 (42.4)	196 (41.2)	79 (45.9)	
Male	373 (57.6)	280 (58.8)	93 (54.1)	
**Race/Ethnicity**				0.29^c^
Hispanic, any race	18 (2.8)	11 (2.3)	7 (4.1)	
Non-Hispanic Black	51 (7.9)	38 (8.0)	13 (7.6)	
Non-Hispanic White	565 (87.2)	419 (88.0)	146 (84.9)	
Other	14 (2.2)	8 (1.7)	6 (3.5)	
Insurance Type*				***<0.001***^b^
Public (Medicare/caid)	186 (30.7)	121 (27.4)	65 (39.4)	
Private/Other	420 (69.3)	320 (72.6)	100 (60.6)	
**Zip code median income quartile (Q)**				0.23^b^
Q1 ($1–45,999)	119 (19.3)	78 (17.3)	41 (24.6)	
Q2 ($46,000–58,999)	201 (32.5)	151 (33.5)	50 (29.9)	
Q3 ($59,000–78,999)	198 (32.0)	149 (33.0)	49 (29.3)	
Q4 ($79,000+)	100 (16.2)	73 (16.2)	27 (16.2)	

Data presented on HCT recipients. Karnofsky performance score data not available for all subjects.

Statistically significant *p*-values are in bold.

*Data not available for all subjects. Missing values: Age = 1; Race/Ethnicity = 10; Insurance type = 42; Race/Ethnicity = 10.

Statistics presented as Median [25th, 75th percentile], *N* (column %).

*p*-values:

^a^Wilcoxon Rank Sum test.

^b^Pearson’s chi-square test.

^c^Fisher’s Exact test (two-tailed).

Finally, we assessed whether there were differences in diagnosis by demographics (Table 5). Younger median age was associated with leukemia and lymphoma diagnoses versus myeloma/amyloidosis, MDS/MPN, and myelofibrosis (pairwise comparison, *p* < 0.05). Sex was associated with diagnosis (*p* = 0.02) such that males comprised a higher percentage of lymphoma versus leukemia diagnoses (pairwise comparison, *p* < 0.05). Race/ethnicity was also associated with diagnosis (*p* = 0.01). There was no significant association between diagnoses and insurance type (*p* = 0.24) or quartiles of median household income (*p* = 0.45).

**Table 5:** Association between most common diagnoses for HCT recipients by demographics.

		Diagnosis				
Factor	Total (*N* = 629)	Leukemia (*N* = 171)	Myeloma/amyloidosis (*N* = 221)	MDS/MPN/myelofibrosis (*N* = 63)	Lymphoma (*N* = 174)	*p*-value
Age at most recent assessment*	59.9 [50.2, 66.3]	59.0 [45.2, 64.7]^e,f^	61.6 [54.5, 67.2]^d,g^	63.9 [57.6, 67.8]^d,g^	56.9 [47.4, 64.3]^e,f^	***<0.001***^b^
**Sex**						***0.02***^c^
Female	265 (42.1)	82 (48.0)^h^	98 (44.3)	29 (46.0)	56 (32.2)^e^	
Male	364 (57.9)	89 (52.0)	123 (55.7)	34 (54.0)	118 (67.8)	
**Race/Ethnicity**						***0.01***^a^
Hispanic, any race	15 (2.4)	5 (2.9)	5 (2.3)	0 (0)	5 (2.9)	
Non-Hispanic Black	49 (7.8)	7 (4.1)	31 (14.0)	4 (6.4)	7 (4.0)	
Non-Hispanic White	553 (87.9)	155 (90.6)	182 (82.4)	58 (92.1)	158 (90.8)	
All other groups	12 (1.9)	1 (2.3)	3 (1.4)	1 (1.6)	4 (2.3)	
Insurance Type*						0.24^c^
Public (Medicare/caid)	181 (30.7)	43 (28.5)	75 (34.9)	21 (34.4)	42 (25.9)	
Private/Other	408 (69.3)	108 (71.5)	140 (65.1)	40 (65.6)	120 (74.1)	
**Zip code median household income quartile (Q)**						0.45^c^
Q1 ($1–45,999)	116 (19.3)	28 (17.9)	51 (23.5)	8 (13.6)	29 (17.2)	
Q2 ($46,000–58,999)	196 (32.6)	58 (37.2)	58 (26.7)	21 (35.6)	59 (34.9)	
Q3 ($59,000–78,999)	191 (31.8)	47 (30.1)	73 (33.6)	21 (35.6)	50 (29.6)	
Q4 ($79,000+)	98 (16.3)	23 (14.7)	35 (16.1)	9 (15.3)	31 (18.3)	

Data presented on HCT recipients. Diagnosis data not available for all subjects.

Statistically significant *p*-values are in bold.

*Data not available for all subjects. Missing values: Age at assessment = 1; Quartile, median income by zip = 31; Insurance type = 40.

Statistics presented as Median [P25, P75], *N* (column %).

*p*-values:

^a^ANOVA.

^b^Kruskal–Wallis test.

^c^Pearson’s chi-square test.

^d^Significantly different from Leukemia.

^e^Significantly different from Myeloma/amyloidosis.

^f^Significantly different from MDS/MPN/myelofibrosis.

^g^Significantly different from Lymphoma.

Post-hoc pairwise comparisons were done using Bonferroni adjustment.

## Discussion

Patients from historically marginalized racial/ethnic groups, those residing in disadvantaged neighborhoods, and who are publicly insured are less likely to receive HCT even after being evaluated at an HCT center. A strong income bias in PACT scores raises concern as to whether the psychosocial assessment is systematically impeding access to HCT in poorer populations. Encouragingly, race/ethnicity and insurance type were not associated with PACT scores. This contrasts with a previous study by Hong et. al. limited to adult allogeneic transplants which reported patients of the White race were nearly 3 times more likely to have higher PACT scores than those of the non-White race [18]. While differences across race/ethnicity and insurance type may be partially explained by differences in diagnosis and baseline physical health, respectively, we did not find any association between neighborhood income and HCT-CI score, Karnofsky performance score, or diagnosis.

Limitations of this study include the inability to definitively determine the reason patients were not transplanted, the recommended transplant type or diagnoses for those who did not proceed to HCT, and the specific psychosocial barriers contributing to differences in PACT scores. Additionally, the demographics of the assessors were unknown and therefore we were unable to assess concordance or discordance between the race and ethnicity of the assessor and patient. Our cohort was primarily non-Hispanic White, and small cell size among Hispanics and all other groups may have weakened our ability to discern differences using pairwise comparisons across diagnoses. We used a neighborhood-level proxy for individual socioeconomic status and did not have access to more granular measures of adverse social determinants of health that may underly the observed disparities. Of note, the cohort had a high mortality rate, particularly among those who did not receive transplants. Mortality is a biased indicator since patients who were assessed but not transplanted may have died before their transplant whereas the data selection of an initial transplant prevented this competing risk. Lastly, this was a single-center study. While we expect that similar disparities exist across centers, multi-institutional studies are needed to support our results.

This study had several strengths. Foremost, our dataset uniquely provided information on patients who did not proceed with HCT. We also had access to an institutionally uniform psychosocial scoring system for patients receiving HCT. The analysis was performed post-2012 when haploidentical HCT became widely available at this institution to limit racial and ethnic disparities in eligibility due to a lack of available donors. This period also avoids the COVID-19 pandemic wherein clinical practices were altered.

There are important donor, recipient, and caregiver considerations in taking a patient to HCT [11]. Survivors of HCT may be cured of their primary disease but suffer other serious complications, such as infertility, graft-versus-host disease, secondary malignancies, financial toxicity, and cognitive impairments [27]. Thus a rigorous eligibility assessment is warranted.

Other rating scales used to determine transplant eligibility such as the HCT-CI, have discrete criteria to determine the severity of comorbidities [28]. For example, a patient with a body mass index (BMI) > 35 kg/m^2^ receives an additional 1 point on the HCT-CI [28]. This helps ensure uniform evaluation of BMI and provides a target BMI to help patients achieve prior to transplant. Contrastingly, the parameters of psychosocial assessments are more subjective. A survey study of HCT professionals given patient vignettes with psychosocial information found a complete lack of unanimity in eligibility determinations [29]. Respondents’ determinations were found to be primarily based on their perceived severity of the psychosocial issue [29].

Inarguably, psychosocial factors have the potential to affect HCT outcomes. For example, patients with crowded living spaces during an extremely immunocompromised state are more likely to have infectious complications [30]. Patients without financial stability are less likely to have a full-time caregiver who can take leave from work [31]. However, instead of making these conditions prohibitive to care, efforts should be devoted to help remove modifiable barriers.

Recently, the American Society for Transplantation and Cellular Therapy and the National Marrow Donor Program formed the ACCESS initiative aimed to address recurring inequities in access and outcomes from HCT [32]. Among its initiatives, a poverty committee was tasked to identify psychosocial and financial resources available for HCT candidates [32]. This initiative represents an opportune way to use PACT scoring to collect psychosocial data at the time of HCT assessment and develop an individualized resource toolkit to address psychosocial concerns that may influence transplant outcomes. Future directions should prospectively review whether provisions of psychosocial support services implemented pre-HCT improve PACT scores among socioeconomically disadvantaged populations and whether an improvement in PACT scores leads to more equitable access to transplants. In an era of expanding donor pools, indications for transplant, reduced intensity conditioning, and cellular therapy [33], it is the duty of our HCT centers to ensure these assessments are used to improve outcomes rather than limit access to transplants for vulnerable populations.

## Data Availability

The individual-level data underlying this article cannot be shared due to the privacy of those who participated in the study. Summary-level data without individual-level data are available from the corresponding author on reasonable request.
